# Evaluation of cyclic fatigue and bending test for different Nickle-Titanium files

**DOI:** 10.1371/journal.pone.0290744

**Published:** 2023-08-25

**Authors:** Farah Ramadan, Ammar AbuMostafa, Dalia Alharith

**Affiliations:** Department of Restorative Dentistry, Riyadh Elm University, College of Dentistry, Riyadh, Saudi Arabia; University of Puthisastra, CAMBODIA

## Abstract

**Aim:**

To compare cyclic fatigue resistance and bending for three different nickel-titanium (NiTi) rotary files.

**Materials and methods:**

A sample of 90 NiTi instruments size (25.06) was divided into three groups with 30 files in each: Race Evo files (FKG Dentaire, Switzerland); Tia Tornado Blue files (TiaDent Inc., Texas- USA); One Curve files (Micro-Mega, France). Then each group was subdivided into two groups with 15 files in each; a bending test was performed for one group, and a dynamic cyclic fatigue test at body temperature was performed for the other group. Files fractured by cyclic fatigue were randomly picked from all tested groups for Scanning Electron Microscopy (SEM) (Jeol, Tokyo, Japan). In addition, the test included measuring the broken part of the files tested using (Electronic Micrometre Calliper with LCD Screen, Inch and Millimetre Conversion, Adoric- Taiwan). Data were statistically analyzed using a one-way ANOVA with Tukey-HSD post hoc test.

**Results:**

It was found that maximum load [gf] was less (meaning more flexible) in the group of Tia Tornado Blue with a statistically significant difference in comparison with Race Evo and One Curve. The number of cycles to fracture (NCF) in the Race Evo group was significantly higher than the groups of One Curve and Tia Tornado Blue.

**Conclusions:**

Within the limitation of the study, it could be concluded that Race Evo files were more resistant to cyclic fatigue fracture and Tia Tornado Blue files were more flexible compared to the other tested files.

## Introduction

Instrumentation is the fundamental aspect of root canal preparation. Throughout the years, root canal files have evolved and seen a complete makeover after the introduction of NiTi which has been used in endodontics for almost 25 years due to its flexibility and elasticity. The use of NiTi instruments had overcome the shortcomings and disadvantages of stainless-steel instruments [1]. This is mainly due to the property of NiTi files which can more readily maintain the appropriate centrality of the canal, thus helping in achieving a better outcome [2]. Although NiTi has shown properties that are more beneficial than its predecessor, this material too is not immune to fracture, as it will break if its ultimate strength is surpassed [3].

The most common reason for the fracture of the endodontic file is cyclic fatigue or torsional overload. This leads to metal fatigue which ultimately leads to the fracture of files. In a curved canal, the file rotates freely, which causes the inside portion of the file to compress and the outer portion of the file to stretch. When this process repeats several times, fracture occurs in the files due to metal fatigue [4]. Although NiTi files are structured strategically to withstand several hundreds of such flexural cycles before fracturing, fractures can still occur [5]. Since cyclic fatigue resistance is the required parameter in the NiTi rotary file, several studies have been conducted to find its impact on clinical use with variations in the prevalence of cyclic fatigue [6–8]. Moreover, in many previous studies, cyclic fatigue resistance was studied at room temperature and at static rather than in dynamic motion; however, cyclic fatigue resistance should be studied at the body temperature and both static and dynamic motion. Thus, it is very important to view the conditions of the experiments when comparing different studies [9–13].

Another factor that must be assessed with cyclic fatigue is bending stiffness. The greater the flexibility of the files, the better their bending stiffness becomes. Previous studies reported that, when the file is exposed to heat treatment, it may improve cyclic fatigue resistance. However, this may have a negative effect on bending stiffness [14,15]. Several systems of files have been introduced into the market with thermomechanical treatments. Three systems among them are a novice and being used in endodontics. They are Race Evo files (FKG Dentaire, Switzerland), Tia Tornado Blue files (TiaDent Inc., Texas-USA), and One Curve (Micro-Mega, France). All of these heat-treated NiTi rotary file systems have comparable physical and mechanical properties and are more flexible and resistant to cyclic fatigue than traditional NiTi rotary systems. At the time of conducting this research, there was no published study comparing the cyclic fatigue resistance and bending for the files tested in this study. This study aims to compare cyclic fatigue resistance and bending for three different NiTi rotary files: Race Evo files, Tia Tornado Blue files, and One Curve (Micro-Mega, France).

## Materials and methods

This is an in vitro experimental study comparing the cyclic fatigue resistance and bending for three different NiTi rotary files. Ethical approval was obtained from Riyadh Elm University, Riyadh, Saudi Arabia (Registration no. FPGRP/2021/600/511IRB and Approval no. FPGRP/2021/600/511/494). The sample size for the present study was calculated using G power 3.0.10 version software. The alpha error was fixed at 5% and the power of the study was fixed at 90%. A total of 90 NiTi files (size 25 taper 0.06 and length 25 mm), were divided into three groups: Group #1 (n = 30): Race Evo files; Group #2 (n = 30): Tia Tornado Blue files; Group #3 (n = 30): One curve files. Each group was subdivided into 2 subgroups (n = 15), one for cyclic fatigue and one for bending resistance. The instruments were inspected under a dental microscope (Zumax OMS2350, China) at high magnification for defects or deformities. Defective files were replaced with new files.

### Dynamic cyclic fatigue test

The cyclic fatigue test was conducted using a dynamic cyclic fatigue device. To simulate clinical conditions, the test has been done in a dynamic model at body temperature. A dynamic test device consists of a reciprocating cyclic linear dc gear electric motor (Mainland, Guangdong, China) fixed by a portable tripod (Promate, Shenzhen, China) to simulate axial dynamic motion, set at a reproducible position. A rotary (6:1 reduction) handpiece (Dentsply Maillefer, Ballaigues, Switzerland) powered by a torque-controlled electric motor (X-smart plus, Dentsply Maillefer, Ballaigues, Switzerland) then, connected to the reciprocating gear electric motor by a plastic connector. Furthermore, a digital water bath (DXY, China) has been used occupied with normal saline at body temperature (37°±1) to conduct the test similar to the clinical condition.

**Fig 1:**
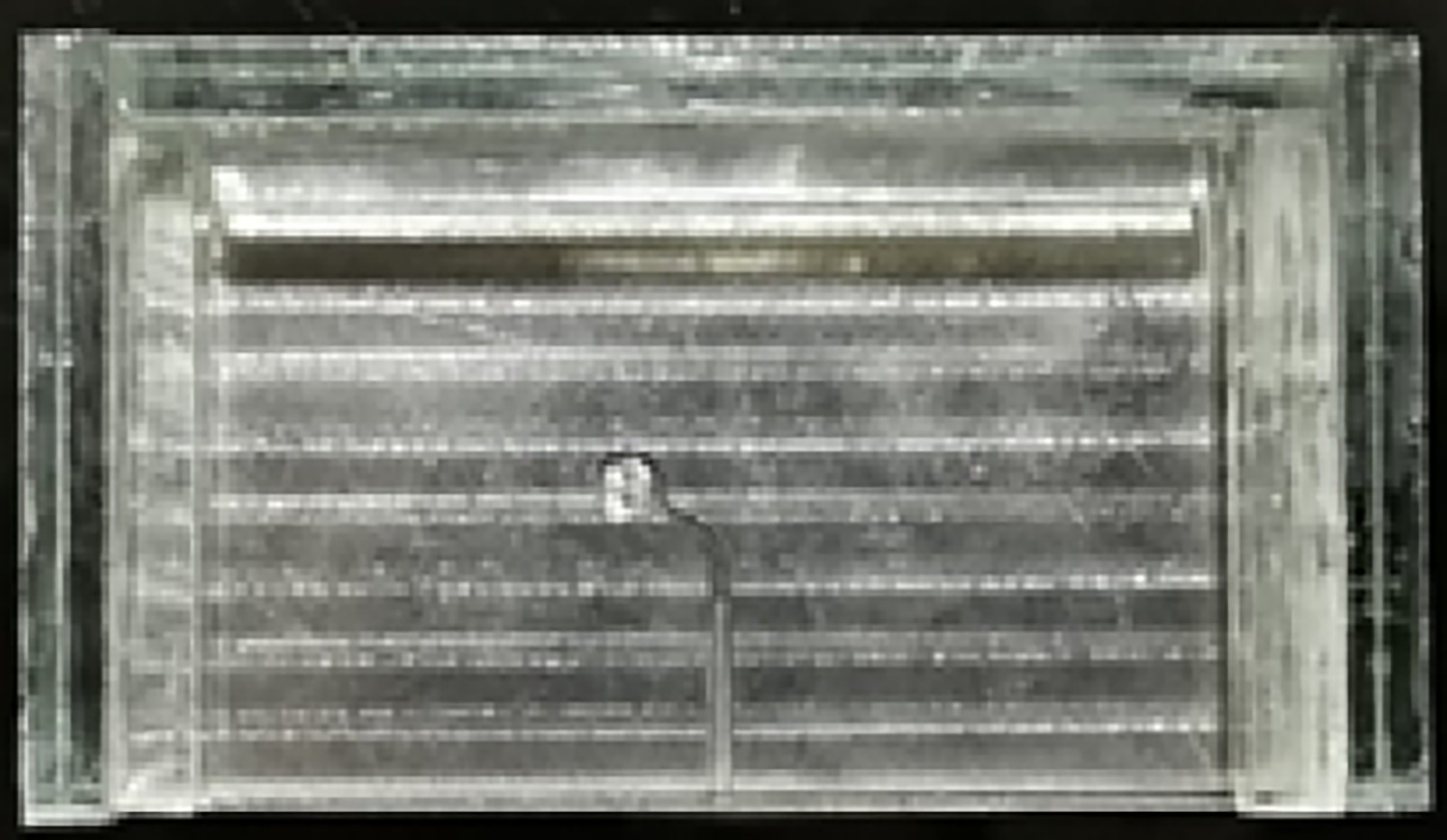
The stainless-steel plate.

An artificial curved canal was made within a stainless-steel plate according to a recommendation of Pruett et al. [16] using two parameters: Angle of curvature and Radius of curvature covered with a tempered transparent glass plate that permits the direct observation of the moment of fracture with a curvature of 60° and 5-mm radius, an inner diameter of 1.5 mm, a total length of 25 mm, and the maximum curvature at 5 mm from the apical tip of the canal. The artificial canal was manufactured by reproducing the exact size and taper of the instruments to be tested, thus providing the instrument with a suitable trajectory that follows exactly the parameters of the curvature chosen for the experiment (Fig 1).

**Fig 2:**
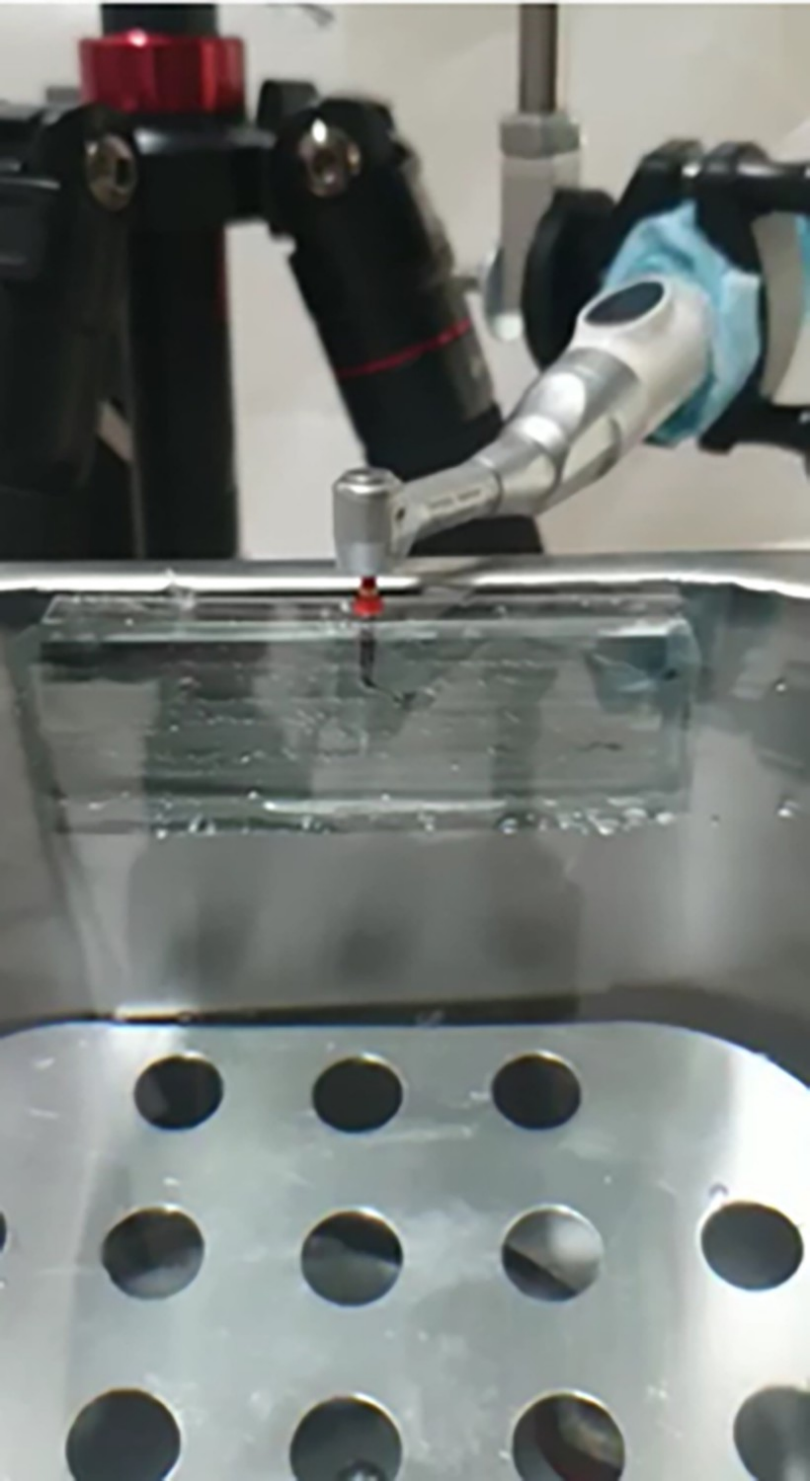
Set up of the dynamic cyclic fatigue test.

The plate was fixed inside a digital thermostat water bath (DXY, China). The container was filled with normal saline adjusted at body temperature (37°±1) to simulate the clinical setting. Each file was introduced into the artificial canal up to the end of the artificial canal after the file begins rotating. The files were powered at the torque and speed according to the manufacturer’s instructions: Race Evo files (FKG Dentaire, Switzerland): speed of 850 rpm and torque of 1.5 N/cm; Tia Tornado Blue files (TiaDent Inc., Texas-USA): speed of 350 rpm and torque of 1.5 N/cm; One Curve files (Micro-Mega, France): speed of 350 rpm and torque of 1.5 N/cm (Fig 2).

The instruments were rotated freely within a metal block filled with silicone lubricant (CRC Inc., Warminster, U.S.A) to reduce friction and heat production. The instruments were rotated until a fracture occurred visually and/or audibly. To avoid human error, video recording was performed simultaneously with a digital camera (Canon Inc., Taiwan), and the recordings were then observed to cross-check the time of file separation. The time to fracture was registered in seconds. The NCF was then calculated by multiplying the number of seconds in rotation with the speed of rotation of the motor (rpm)/60 seconds.

### Measuring the broken parts

In addition, the test included measuring the broken part of the files tested using (Electronic Micrometre Calliper with LCD Screen, Inch and Millimetre Conversion, Adoric- Taiwan), as an additional measure of comparison between different types of files.

### Bending resistance test

The bending resistance test was conducted according to the ISO 3630–1 specification by using a universal testing machine (Model no.5967 Instron, Canton, MI, USA) at room temperature and a crosshead speed of 15 mm/min. This instrument is evaluated by clamping 3 mm of its tip in a chuck and applying an angular deflection of 45°. The machine was supported by a computer program that guarantees a correct and reliable recording value recorded in gram-force (gf). The maximum load was recorded and statistically analyzed.

### Scanning electron microscopy (SEM)

Nine files fractured by cyclic fatigue were randomly picked from all tested groups for SEM (Jeol, Tokyo, Japan) analysis to look for the topographic pattern of the fractured files. Samples were cleaned of debris in an ultrasonic cleaner for 6 minutes using 30% ethanol and were then mounted in a vertical position using a custom holder.

### Statistical analysis

Descriptive statistics of the cyclic fatigue and bending test measurements were performed. The comparison between the cyclic fatigue resistance and bending test for the three different titanium rotary files has been analyzed using a one-way ANOVA along with Tukey-HSD post-hoc. A p-value cut-off point of 0.05 at 95% CI was used to determine statistical significance. All data analyses were performed using Statistical Packages for Software Sciences (SPSS) version 26 (Armonk, New York, IBM Corporation, USA).

## Results

The mean ± sd of maximum load [gf], number of seconds, NCF, and broken part [mm] for all files are listed in (Table 1). Maximum load represents bending test and flexibility; the highest the value, the least the flexibility. NCF represents cyclic fatigue resistance; the highest the value, the highest cyclic fatigue resistance.

**Table 1:** Descriptive statistics of the cyclic fatigue and bending test.

Variables	Mean (for all files)	SD	Median	Min	Max
**Maximum load [gf]**	515.5	66.8	525.6	355.8	626.5
**Number of seconds**	215.9	82.7	192.0	48.0	376.0
**NCF**	1681.8	581.7	1761.7	280.0	3102.5
**Broken part (mm)**	4.97	1.76	4.12	3.01	8.42

The mean values of maximum load were significantly less (p<0.001) in Tia Tornado. NCF was statistically significantly higher (p<0.001) in Race Evo. The mean value of the number of seconds was statically significantly longer in One Curve (p<0.001), whereas the mean value of the broken part was statistically significantly more in Tia Tornado (p<0.001) (Table 2).

**Table 2:** Comparison of cyclic fatigue resistance and bending test for the three different- titanium rotary files.

Measurement	Instrument			
	One Curve Mean ± SD	Race Evo Mean ± SD	TIA Tornado Mean ± SD	
**Maximum load [gf]**	537.7 ± 24.2	561.6 ± 50.9	447.0 ± 55.0	<0.001 ***
**Number of seconds**	297.3 ± 50.7	152.1 ± 34.3	198.3 ± 77.1	<0.001 ***
**NCF**	1734.4 ± 295.9	2154.3 ± 485.9	1156.6 ± 449.5	<0.001 ***
**Broken part (mm)**	3.69 ± 0.38	3.96 ± 0.61	7.24 ± 0.91	<0.001 ***

^§^ P-value has been calculated using the One-way ANOVA.

Significant at p<0.001 level.

The mean maximum load value was significantly higher in Race Evo (p<0.001). The mean number of seconds was significantly longer in One Curve (p<0.001). The mean of NCF was statistically significantly higher in Race Evo (p<0.001). The mean value of the broken part was statistically significantly longer in Tia Tornado (p<0.001).

When measuring the multiple differences in the mean values of the three different-titanium rotary files by the cyclic fatigue and bending test, significant differences were found in maximum load [gf] between One Curve and Tia Tornado (p<0.001) and between Race Evo and Tia Tornado (p<0.001). In the number of seconds, statistically, significant differences were found between Race Evo and Once Curve (p<0.001), and between Tia Tornado and One Curve (p<0.001). In NCF, statistically significant differences were found between Race Evo and One Curve (p = 0.024), between Race Evo and Tia Tornado (p<0.001), and between Tia Tornado and One Curve (p = 0.001). Finally, in the broken part, statistically significant differences were found between One Curve and Tia Tornado (p<0.001) and between Race Evo and Tia Tornado (p<0.001) (Table 3).

**Table 3:** Post hoc test for the multiple differences of cyclic fatigue and bending test in the three different-titanium rotary files.

Dependent variable	Instrument (I)	Instrument (J)	Mean Difference (I–J)	95% CI	p-value §
**Maximum load [gf]**	One curve	Race Evo	-23.930	-64.26–16.40	0.329
		Tia tornado	90.698	50.37–131.03	**<0.001** ***
	Race Evo	One Curve	23.930	-16.40–64.26	0.329
		Tia tornado	114.628	74.30–154.96	**<0.001** ***
	Tia tornado	One Curve	-90.698	-131.03 –-50.37	**<0.001** ***
		Race Evo	-114.628	-154.96 –-74.30	**<0.001** ***
**Number of seconds**	One curve	Race Evo	145.267	94.85–195.68	**<0.001** ***
		Tia tornado	99.067	48.65–149.48	**<0.001** ***
	Race Evo	One Curve	-145.267	-195.68 –-94.85	**<0.001** ***
		Tia tornado	-46.200	-96.61–4.21	0.078
	Tia tornado	One Curve	-99.067	-149.48 –-48.65	**<0.001** ***
		Race Evo	46.200	-4.21–96.61	0.078
**NCF**	One curve	Race Evo	-419.833	-791.19 –-48.47	**0.024** ***
		Tia tornado	577.884	206.53–949.24	**0.001** ***
	Race Evo	One Curve	419.833	48.47–791.19	**0.024** ***
		Tia tornado	997.718	626.36–1369.08	**<0.001** ***
	Tia tornado	One Curve	-577.884	-949.24 –-206.53	**0.001** ***
		Race Evo	-997.718	-1369.08 –-626.4	**<0.001** ***
**Broken part [mm]**	One curve	Race Evo	-0.263	-0.857–0.331	0.533
		Tia tornado	-3.548	-4.142 –-2.954	**<0.001** ***
	Race Evo	One Curve	0.263	-0.331–0.857	0.533
		Tia tornado	-3.285	-3.879 –-2.691	**<0.001** ***
	Tia tornado	One Curve	3.548	2.954–4.142	**<0.001** ***
		Race Evo	3.285	2.691–3.879	**<0.001** ***

^§^ Post hoc test has been calculated using the Tukey-HSD post-hoc.

*** Significant at p<0.001 level.

**Fig 3:**
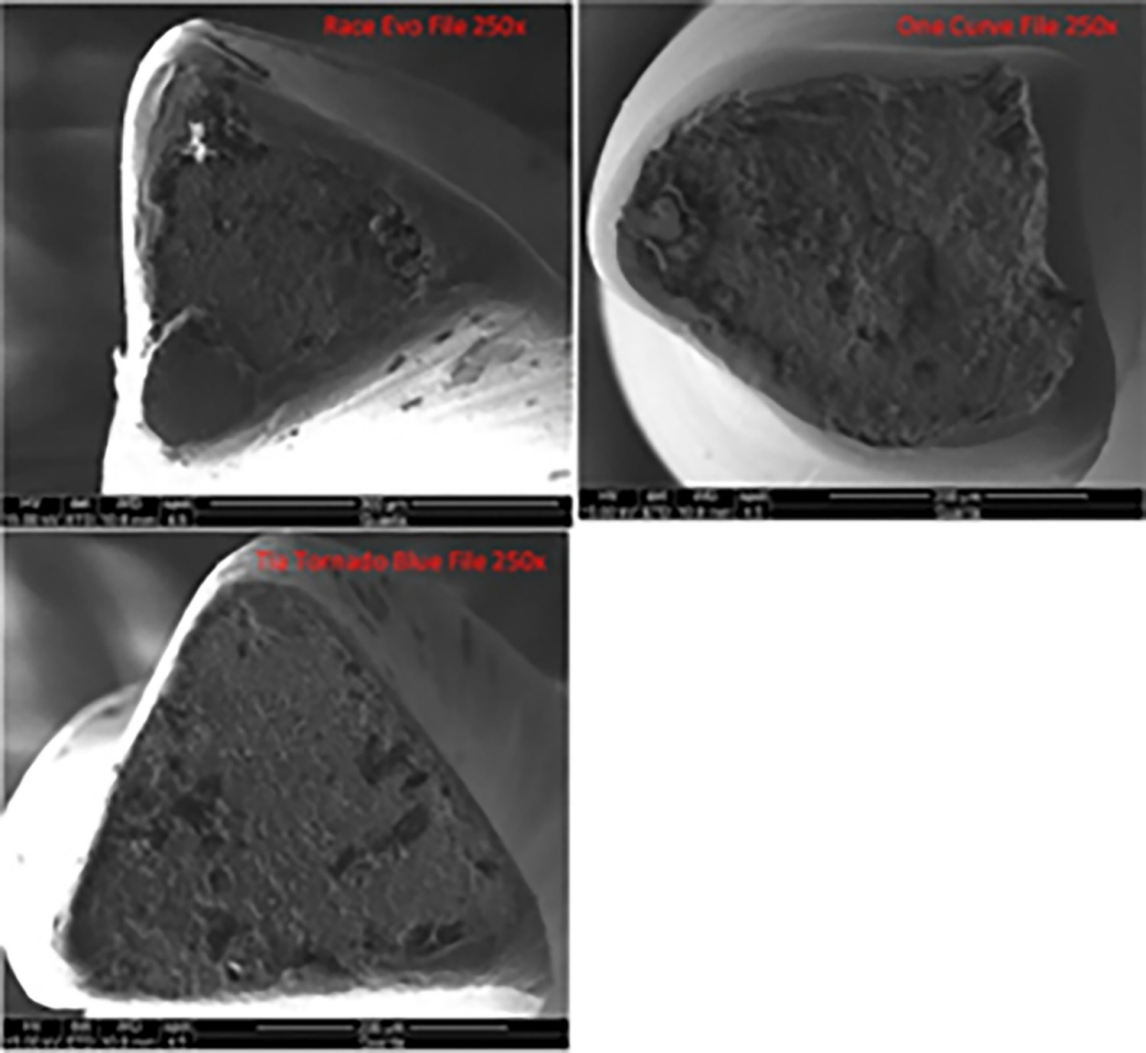
SEM images for different files.

The SEM images show the fractured surfaces of instruments after cyclic fatigue, confirming a predominantly ductile mode of fracture. Propagation points at the surface, striations, and dimples are obvious on the surface (Fig 3).

## Discussion

The present study used file systems that were recently introduced to the endodontic practice to assess their cyclic fatigue resistance and bending resistance. As per the review of the literature, this is one of the first studies to be conducted comparing the cyclic fatigue and bending resistance for One Curve, Race Evo, and Tia Tornado blue files. We used a dynamic cyclic fatigue test rather than the static fatigue test to assess cyclic fatigue resistance. This is because studies utilizing static fatigue tests reported their inherent disadvantages and the dynamic test mimics the clinical situation more closely than the static fatigue test [17,18]. Dynamic cyclic fatigue test provides pecking movement, thus helping in mimicking the clinical setting. This test axial movement provided allows the distribution of the load along the entire shaft of the file avoiding a localized load in a single point which increases the life span of the file. This has been proved in the studies done previously [19,20].

Instead of considering the human teeth for the experiment, the present study has taken into consideration stainless steel canals, since the standardization of the canal in extracted human teeth is a difficult task and subject to standardization. Furthermore, to allow the comparison of results and match the study results with those of previous studies, the present study considered a 60-degree curvature angle and a 5-mm curvature radius for the stainless-steel canal [21,22].

To simulate the clinical setting, the current study used a dynamic test model at a body temperature (of 37°C). Some authors have shown that body temperature drastically affects the flexural resistance of NiTi files because it is capable of modifying the transformation temperatures of the NiTi [12,13,23,24]. The present study utilized the universal testing machine to study the bending resistance which is commonly employed by many previous studies, thus allowing the comparison of the present study results with those of previous studies [15,25].

In the present study, the mean maximum load was significantly less in Tia Tornado files, meaning they are more flexible than the other tested files. NCF was statistically significantly higher in Race Evo, while the mean number of seconds was statistically significantly longer in One Curve. The mean length of the broken part was statistically significantly more in Tia Tornado. Though all are heat-treated alloys, NCF for Race Evo was significantly higher than One Curve and Tia Tornado. This could be attributed to the electropolishing of Race Evo files. Regarding the NCF values, the results for One Curve (1734.4 ± 295.9) are similar to the values of Ghahramani et al. (1333±234) [23]. On the other hand, the NCF for One Curve in another study (301 ± 38) [26] was significantly less than the results from this present study. This could be due to the difference in the mode of cyclic fatigue test used, as the present study used a dynamic model, while the study of Serafin et al. [26] used a static model.

Despite the similarity in heat treatment and geometry of the files used in the present study, it has been found that the maximum load [gf] for the files of Tia Tornado was significantly less, meaning more flexible than Race Evo and One Curve. Up to the knowledge of the researchers, there is no published study about the bending test of Tia Tornado blue files, making it difficult to justify and compare. The relationship between bending resistance and cyclic fatigue resistance is contradictory in the literature. Some studies found that flexible instruments present a higher number of cycles to fracture [27–29]. On the other hand, some studies are consistent with ours in which no relationship was found between bending and cyclic fatigue resistance [30–32].

SEM was used in this investigation due to its popular influence in endodontic literature as it attains an advantageous ability in detecting the extent or mode of the fracture. The present study has some limitations. The study is done in an in-vitro stainless steel plate model in a water bath to simulate body temperature. However, the conditions during the root canal treatment for clinical cases may differ due to the different properties of the human dentine compared to the metal blocks. The present study utilized a single defined curvature with a specific curvature design and arc length. Thus, the result obtained may not apply to different conditions. Lastly, this study used a specific length and diameter of the instrument to standardize and compare the results. The results may vary when these dimensions are altered.

## Conclusion

Within the limitation of the study, it can be concluded that Race Evo files were significantly more resistant to cyclic fatigue compared to One Curve and Tia Tornado and Tia Tornado files were significantly more flexible compared to One Curve and Race Evo. A more clinically simulated model (curvature and anatomical variation) can be used in the future that resembles the real root dentine characteristics.

## Supporting information

S1 Dataset (DOCX)
