# Supplementary material for: Evaluation of cyclic fatigue and bending test for different Nickle-Titanium files
Source: PLoS One. 2023 Aug 25;18(8):e0290744. doi: 10.1371/journal.pone.0290744 (PMC10456180; doi:10.1371/journal.pone.0290744)
Supplement: S1 Dataset — (DOCX) [file pone.0290744.s001.docx]

**Data set**

| **Cyclic fatigue collection sheets** | | | |
| --- | --- | --- | --- |
| **Samples** | Tia Tornado | | |
|  | 𝑛𝑢𝑚𝑏𝑒𝑟 𝑜𝑓 𝑠𝑒𝑐𝑜𝑛𝑑𝑠 | NCF* | Broken part |
| **1** | 48 | 280 | 6.91 |
| **2** | 161 | 939.166667 | 7.99 |
| **3** | 199 | 1160.83333 | 7.21 |
| **4** | 84 | 490 | 7.89 |
| **5** | 300 | 1750 | 7.12 |
| **6** | 175 | 1020.83333 | 7.74 |
| **7** | 180 | 1050 | 8.06 |
| **8** | 178 | 1038.33333 | 7.72 |
| **9** | 213 | 1242.5 | 8.06 |
| **10** | 169 | 985.833333 | 6.91 |
| **11** | 182 | 1061.66667 | 6.92 |
| **12** | 299 | 1744.16667 | 8.42 |
| **13** | 184 | 1073.33333 | 5.54 |
| **14** | 272 | 1586.66667 | 5.25 |
| **15** | 330 | 1925 | 6.91 |
|  | Average | 1156.6 |  |

| **Cyclic fatigue collection sheets** | | | |
| --- | --- | --- | --- |
| **Samples** | Race Evo | | |
|  | 𝑛𝑢𝑚𝑏𝑒𝑟 𝑜𝑓 𝑠𝑒𝑐𝑜𝑛𝑑𝑠 | NCF** | Broken part |
| **1** | 160 | 2266.7 | 4.56 |
| **2** | 110 | 1558.3 | 3.52 |
| **3** | 111 | 1572.5 | 4.74 |
| **4** | 126 | 1785.0 | 4.40 |
| **5** | 126 | 1785.0 | 3.56 |
| **6** | 219 | 3102.5 | 4.93 |
| **7** | 129 | 1827.5 | 4.33 |
| **8** | 128 | 1813.3 | 3.06 |
| **9** | 130 | 1841.7 | 3.14 |
| **10** | 158 | 2238.3 | 3.08 |
| **11** | 187 | 2649.2 | 4.26 |
| **12** | 215 | 3045.8 | 3.54 |
| **13** | 160 | 2266.7 | 3.90 |
| **14** | 155 | 2195.8 | 4.35 |
| **15** | 167 | 2365.8 | 4.01 |
|  | Average | 2154.3 |  |

| **Cyclic fatigue collection sheets** | | | |
| --- | --- | --- | --- |
| **Samples** | One Curve | | |
|  | 𝑛𝑢𝑚𝑏𝑒𝑟 𝑜𝑓 𝑠𝑒𝑐𝑜𝑛𝑑𝑠 | NCF* | Broken part |
| **1** | 302 | 1761.7 | 3.48 |
| **2** | 317 | 1849.2 | 4.12 |
| **3** | 238 | 1388.3 | 4.11 |
| **4** | 293 | 1709.2 | 4.12 |
| **5** | 244 | 1423.3 | 3.42 |
| **6** | 356 | 2076.7 | 3.39 |
| **7** | 376 | 2193.3 | 4.05 |
| **8** | 313 | 1825.8 | 4.12 |
| **9** | 277 | 1615.8 | 3.11 |
| **10** | 192 | 1120.0 | 3.78 |
| **11** | 372 | 2170.0 | 3.01 |
| **12** | 310 | 1808.3 | 3.60 |
| **13** | 306 | 1785.0 | 3.60 |
| **14** | 310 | 1808.3 | 4.04 |
| **15** | 254 | 1481.7 | 3.48 |
|  | Average | 1734.4 |  |

| **Bending test collection sheets** | | | |
| --- | --- | --- | --- |
| **Endodontic Files Race Evo** | | | |
| **Samples** | **Maximum Load [gf]** | **Tensile stress at Maximum Load [MPa]** | **Extension at Break (Standard) [mm]** |
| **1** | 608.7325 | 1.22 | 42.13797 |
| **2** | 590.8132 | 1.18 | 42.14861 |
| **3** | 584.0916 | 1.17 | 42.15062 |
| **4** | 626.49 | 1.25 | 42.18809 |
| **5** | 525.5987 | 1.05 | 42.1006 |
| **6** | 621.8057 | 1.24 | 42.22556 |
| **7** | 544.8232 | 1.09 | 42.22561 |
| **8** | 558.4593 | 1.12 | 42.18809 |
| **9** | 440.9996 | 0.88 | 42.18809 |
| **10** | 503.3241 | 1.01 | 42.2004 |
| **11** | 523.618 | 1.05 | 42.20336 |
| **12** | 569.9263 | 1.14 | 42.20737 |
| **13** | 544.6604 | 1.09 | 42.20065 |
| **14** | 620.4973 | 1.24 | 42.18809 |
| **15** | 560.705 | 1.12 | 42.2131 |

| **Coefficient of variation** | 0.087484241 | 0.087020049 | 0.000804541 |
| --- | --- | --- | --- |
| **Maximum** | 626.49 | 1.25 | 42.22561 |
| **Mean** | 561.6363267 | 1.123333333 | 42.184414 |
| **Median** | 560.705 | 1.12 | 42.18809 |
| **Minimum** | 440.9996 | 0.88 | 42.1006 |
| **Range** | 185.4904 | 0.37 | 0.12501 |
| **Standard deviation** | 49.1343278 | 0.097752522 | 0.033939086 |

| **Bending test collection sheets** | | | |
| --- | --- | --- | --- |
| **Endodontic Files Tia Tornado** | | | |
| **Samples** | **Maximum Load [gf]** | **Tensile stress at Maximum Load [MPa]** | **Extension at Break (Standard) [mm]** |
| **1** | 399.0645 | 0.8 | 42.17564 |
| **2** | 474.8056 | 0.95 | 42.11321 |
| **3** | 522.6186 | 1.04 | 42.19306 |
| **4** | 379.2252 | 0.76 | 42.21315 |
| **5** | 493.2786 | 0.99 | 42.16117 |
| **6** | 384.1285 | 0.77 | 42.13812 |
| **7** | 456.6761 | 0.91 | 42.18809 |
| **8** | 355.8258 | 0.71 | 42.08805 |
| **9** | 499.8525 | 1 | 42.1663 |
| **10** | 446.1896 | 0.89 | 42.20065 |
| **11** | 503.2183 | 1.01 | 42.13912 |
| **12** | 516.1996 | 1.03 | 42.11321 |
| **13** | 463.4747 | 0.93 | 42.06308 |
| **14** | 406.1605 | 0.81 | 42.223 |
| **15** | 404.4065 | 0.81 | 42.17564 |
| **Coefficient of variation** | 0.118910333 | 0.118575267 | 0.001070784 |
| **Maximum** | 522.6186 | 1.04 | 42.223 |
| **Mean** | 447.0083067 | 0.894 | 42.156766 |
| **Median** | 456.6761 | 0.91 | 42.1663 |
| **Minimum** | 355.8258 | 0.71 | 42.06308 |
| **Range** | 166.7928 | 0.33 | 0.15992 |
| **Standard deviation** | 53.15390662 | 0.106006289 | 0.045140787 |

| **Bending test collection sheets** | | | |
| --- | --- | --- | --- |
| **Endodontic Files One Curve** | | | |
| **Samples** | **Maximum Load [gf]** | **Tensile stress at Maximum Load [MPa]** | **Extension at Break (Standard) [mm]** |
| **1** | 488.1482 | 0.98 | 42.2006 |
| **2** | 550.9498 | 1.1 | 42.1225 |
| **3** | 537.6907 | 1.07 | 42.15058 |
| **4** | 555.2892 | 1.11 | 42.18879 |
| **5** | 504.5861 | 1.01 | 42.11311 |
| **6** | 550.8398 | 1.1 | 42.16308 |
| **7** | 530.7956 | 1.06 | 42.12898 |
| **8** | 547.1153 | 1.09 | 42.10055 |
| **9** | 562.9644 | 1.12 | 42.15786 |
| **10** | 540.5895 | 1.08 | 42.16308 |
| **11** | 503.0222 | 1 | 42.20054 |
| **12** | 564.0405 | 1.13 | 42.20004 |
| **13** | 541.5839 | 1.08 | 42.2006 |
| **14** | 519.3085 | 1.04 | 42.16303 |
| **15** | 568.6642 | 1.14 | 42.2003 |
| **Coefficient of variation** | 0.043485209 | 0.043246837 | 0.000799084 |
| **Maximum** | 568.6642 | 1.14 | 42.2006 |
| **Mean** | 537.70586 | 1.074 | 42.163576 |
| **Median** | 541.5839 | 1.08 | 42.16308 |
| **Minimum** | 488.1482 | 0.98 | 42.10055 |
| **Range** | 80.516 | 0.16 | 0.10005 |
| **Standard deviation** | 23.38225174 | 0.046447103 | 0.03369222 |
